# Role of Caveolae in the Development of Microvascular Dysfunction and Hyperglycemia in Type 2 Diabetes

**DOI:** 10.3389/fphys.2022.825018

**Published:** 2022-02-18

**Authors:** Yanna Tian, Katie Anne Fopiano, Vijay S. Patel, Attila Feher, Zsolt Bagi

**Affiliations:** ^1^Department of Physiology, Medical College of Georgia, Augusta University, Augusta, GA, United States; ^2^Department of Surgery, Medical College of Georgia, Augusta University, Augusta, GA, United States

**Keywords:** diabetes, endothelial dysfunction, caveolae, caveolin knockout, cyclodextrin, glucose

## Abstract

In type 2 diabetes (T2D) microvascular dysfunction can interfere with tissue glucose uptake thereby contributing to the development of hyperglycemia. The cell membrane caveolae orchestrate signaling pathways that include microvascular control of tissue perfusion. In this study, we examined the role of caveolae in the regulation of microvascular vasomotor function under the condition of hyperglycemia in T2D patients and rodent models. Human coronary arterioles were obtained during cardiac surgery from T2D patients, with higher perioperative glucose levels, and from normoglycemic, non-diabetic controls. The coronary arteriole responses to pharmacological agonists bradykinin and acetylcholine were similar in T2D and non-diabetic patients, however, exposure of the isolated arteries to methyl-β-cyclodextrin (mβCD), an agent known to disrupt caveolae, reduced vasodilation to bradykinin selectively in T2D subjects and converted acetylcholine-induced vasoconstriction to dilation similarly in the two groups. Dilation to the vascular smooth muscle acting nitric oxide donor, sodium nitroprusside, was not affected by mβCD in either group. Moreover, mβCD reduced endothelium-dependent arteriolar dilation to a greater extent in hyperglycemic and obese db/db mice than in the non-diabetic controls. Mechanistically, when fed a high-fat diet (HFD), caveolin-1 knockout mice, lacking caveolae, exhibited a significantly reduced endothelium-dependent arteriolar dilation, both *ex vivo* and *in vivo*, which was accompanied by significantly higher serum glucose levels, when compared to HFD fed wild type controls. Thus, in T2D arterioles the role of caveolae in regulating endothelium-dependent arteriole dilation is altered, which appears to maintain vasodilation and mitigate the extent of hyperglycemia. While caveolae play a unique role in microvascular vasomotor regulation, under the condition of hyperglycemia arterioles from T2D subjects appear to be more susceptible for caveolae disruption-associated vasomotor dysfunction and impaired glycemic control.

## Introduction

Type 2 diabetes mellitus (T2D) is associated with impaired vascular endothelial function in resistance arteries (Nitenberg et al., 1998; Schram and Stehouwer, 2005; Serne et al., 2006; Eringa et al., 2013). Intracoronary injection of the endothelium-dependent agonist, acetylcholine (ACh), which normally dilates arteries, causes vasospasm in patients presenting with angina but no occlusive coronary artery disease; this pathological response was found to be strongly associated with diabetes (Kaneda et al., 2006). In isolated coronary resistance arteries dissected from the heart of diabetic patients, studies have shown that ACh or increases in flow and wall shear stress induce vasoconstriction, which is likely to be attributed to impaired nitric oxide (NO)-mediated signaling (Miura et al., 2003; Beleznai et al., 2011; Cassuto et al., 2014). Interestingly, coronary microvessels from patients with diabetes and coronary artery disease may initially develop a compensatory vasomotor response aiming at maintaining adequate tissue perfusion (Szerafin et al., 2006; Phillips et al., 2007). A disturbed vasomotor control in diabetes is not restricted to coronary arteries and it was reported to present in most vascular beds, including skeletal muscle resistance arteries (Steinberg et al., 1994). For example, vasomotor dysfunction of skeletal muscle resistance arteries not only results in a mismatch between blood supply and demand (Mahgoub and Abd-Elfattah, 1998), but it can also limit the overall tissue glucose uptake, thereby contributing to the development of hyperglycemia in T2D (Muris et al., 2013; Stehouwer, 2018). The nature of the mechanisms through which microvascular dysfunction contributes to the development of hyperglycemia remain incompletely understood in T2D.

Caveolae are 50 to 100 nm cell membrane invaginations that are composed of several key structural and scaffolding proteins, including caveolin-1 (Cav-1), which is found most abundantly expressed in adipocytes, epithelial, and vascular endothelial cells (Nathke et al., 1992; Frank et al., 2003). Cell membrane caveolae uniquely regulate physiological functions including those microvascular mechanisms that control tissue perfusion and metabolism. In this process, signaling molecules may act through a direct interaction with Cav-1 within the cell membrane caveolae. The majority of these molecular interactions are thought to be inhibitory, such as binding Cav-1 to endothelial nitric oxide synthase (eNOS) to limit the production of NO (Venema et al., 1997). In contrast, interaction with Cav-1 was shown to activate certain signaling molecules, such as the insulin receptor (Yamamoto et al., 1998; Nystrom et al., 1999). Our previous studies revealed that vascular endothelial caveolae and Cav-1 play an important role in the maintenance of microvascular vasomotor function (Feher et al., 2010; Cassuto et al., 2014; Czikora et al., 2015; Dou et al., 2017). We found the intact caveolae structure to be critical in maintaining the availability of the eNOS cofactor tetrahydrobiopterin (BH_4_) to remain in close proximity of eNOS, which becomes compromised in diabetes (Cassuto et al., 2014). We have also shown that Cav-1 delays the recycling of type 1 angiotensin receptors to the cell membrane surface, which protects against a sustained receptor activation and vasoconstriction by angiotensin 2 in high-fat diet induced T2D (Czikora et al., 2015). Collectively, these studies indicated that Cav-1 and caveolae play an important physiological role in regulating microvascular homeostasis both under normal and diabetic conditions.

A previous study by Cohen et al. has shown that Cav-1 knockout mice on a normal chow diet displayed higher glucose levels in an insulin tolerance test (Cohen et al., 2003). The Cav-1 knockout mice fed a high-fat diet to induce T2D developed postprandial hyperinsulinemia (Cohen et al., 2003). The impact of T2D on caveolae-regulated vasodilator function and its contribution to the development of hyperglycemia remain unknown. In the current study, we raised the possibility that the vasomotor regulatory role of microvascular endothelial caveolae is critically altered in T2D, which may have implications in the control of systemic glucose homeostasis. In order to test this hypothesis, we set out to examine the impact of caveolae-disrupting agent mβCD and the effect of Cav-1 genetic deficiency on endothelium-dependent vasodilator function in patients and rodent models of T2D, both *ex vivo* and *in vivo*.

## Materials and Methods

### Patients

Protocols involving human subjects were approved by the Institutional Review Board at the Medical College of Georgia. Consecutive patients undergoing heart surgery were enrolled in this study, divided into two groups, with or without documented T2D, irrespective of diabetes duration.

### Mouse Models of Type 2 Diabetes

Protocols involving mice were approved by the Institutional Animal Care and Use Committee at the Medical College of Georgia. Twelve week old male db/db mice (BKS.Cg-m + / + Lepr^db^/J) with leptin receptor deficiency and caveolin-1 knockout mice (Cav^TM 1Mls^/J) as well as corresponding heterozygote and wild type controls were obtained from Jackson Laboratories. As previously described (Cassuto et al., 2014; Czikora et al., 2015), 16 weeks of high-fat diet (HFD, 60% of saturated fat, 58Y1, TestDiet, PMI Nutrition) was initiated in 8-week old male wild type C57BL/6 and caveolin-1 knockout mice. Blood pressure (BP) was measured in conscious mice by tail-cuff plethysmography (CODA, Kent Scientific, Torrington, CT, United States). Non-fasting serum glucose was measured by glucose oxidase method.

### Videomicroscopic Assessment of Dilator Function of Isolated and Pressurized Human and Mouse Resistance Arteries

Videomicroscopy was used to assess vasodilator function of the isolated and pressurized (70 mmHg) human coronary arterioles and mouse skeletal muscle arterioles (100 μm in diameter), as described previously (Szerafin et al., 2006; Fulop et al., 2007; Beleznai et al., 2011; Cassuto et al., 2013, 2014; Feher et al., 2013). Briefly, coronary arterioles were dissected from the right atrial appendages obtained from patients undergoing heart surgery. Mice were anesthetized with an intraperitoneal injection of pentobarbital sodium (50 mg/kg) and under deep anesthesia, the aorta and muscle gracilis muscle were removed. Euthanasia was performed by intraperitoneal injection of sodium pentobarbital (150 mg/kg). Mouse skeletal muscle arterioles were dissected from the m. gracilis. Arteriole dilation was measured in response to endothelium-dependent agonists, bradykinin (Sigma, in human coronary arteries) and acetylcholine (ACh, Sigma, in human coronary and mouse skeletal muscle arterioles) or the direct NO donor, sodium nitroprusside (SNP, Sigma, both in human and mouse). To investigate the role of caveolae, arterioles were pre-incubated with methyl-β-cyclodextrin (mβCD, Sigma, 2 × 10^–3^ M, for 60 min), which is known to disrupt caveolae (Feher et al., 2010; Cassuto et al., 2014).

### Intravital Microscopy of the Mouse Cremaster Muscle

After overnight fasting, mice were anesthetized with a subcutaneous injection of sodium pentobarbital (50 mg/kg). A constant level of anesthesia was maintained throughout the experiments by the subcutaneous injection of supplemental doses (20% of the original dose) of the anesthetic agent every 30–45 min. The trachea was cannulated to facilitate respiration. The left cremaster muscle was exposed through a midline scrotal incision, as described previously (Hohn et al., 2004). An anesthetized mouse was placed on a platform and the cremaster muscle, with nerves and vessels intact, was spread over a heated, transparent pedestal. The whole preparation was then placed on the x-y stage of a microscope (Nikon Eclipse, FN1) and 35°C Krebs buffer was perfused continuously over the muscle at a constant flow rate (2 mL/min). After the surgical procedure, the preparation was allowed to equilibrate for at least 30 min before the start of the experimental protocol. During an incubation period of 30 min, a spontaneous tone developed in the cremaster muscle arterioles. We studied third- and fourth-order cremaster muscle arterioles with internal diameters of ∼20 μm. The arteriolar responses to cumulative concentrations of acetylcholine (0.01–1 μM) and SNP (1–10 μM) were administered (100 μL in a bolus) topically to the surface of the cremaster muscle and the changes in arteriolar diameter were recorded. Images were collected with a CCD camera and were recorded on a DV recorder. The internal arteriolar diameters were measured offline with Image J, by a blinded independent investigator.

### Immunofluorescence Microscopy

Atrial appendages from non-T2D and T2D patients were fixed in 4% paraformaldehyde overnight (at 4°C) and paraffin embedded. Consecutive sections were cut (8 μm thick) and blocked with normal donkey serum (1 h) and immuno-labeled with monoclonal anti-caveolin-1 (Abcam, ab17052, 1:100, overnight, 4°C) antibody. Immuno-fluorescent labeling was performed with corresponding Cy5 secondary antibody (Jackson ImmunoResearch, West Grove, PA, United States). DAPI was used for nuclear staining. For non-specific binding, the primary antibody was omitted. Structured illumination microscopy (SIM-Apotome, AxioImagerM2, CarlZeiss, Jena, Germany) was used for immunofluorescent detection.

### Western Immunoblot

Western immunoblot analysis was carried out as described previously (Beleznai et al., 2011). Briefly, human coronary arterioles were homogenized in radio-immunoprecipitation assay buffer and protein concentration was measured by Bradford assay. Equal amount of proteins were loaded for gel electrophoresis. After blotting, anti-caveolin-1 antibody (Cell Signaling, D46G3, 1:5000) was used for the detection of caveolin-1. Membranes were re-probed with anti-β-actin IgG (1:5000) to normalize for loading variations. Corresponding horseradish peroxidase-labeled secondary antibody was used, and chemiluminescence was visualized autoradiographically.

### Electron Microscopy

The mouse aorta from wild type and caveolin-1 knockout mice were rapidly excised and fixed overnight (at 4°C) in 4% paraformaldehyde, 0.2% glutaraldehyde in 0.1 M sodium cacodylate buffer (pH 7.4), followed by post-fixation in 2% osmium tetroxide (in 0.1 M sodium cacodylate) and stained en bloc with 2% uranyl acetate, subsequently dehydrated and embedded in Epon–Araldite resin. Thin (90 nm) sections were cut with Leica EM UC6 ultramicrotome (Leica Microsystems, Buffalo Grove, IL, United States). Sections were collected on copper grids and stained with uranyl acetate and lead citrate, and observed with a JEM 1230 transmission electron microscope (JEOL United States Inc., Peabody, MA, United States) at 110 kV. Images were collected with an UltraScan 4000 CCD camera and First Light Digital Camera Controller (Gatan Inc., Pleasanton, CA, United States). Similar to our previous study on human coronary arterioles (Cassuto et al., 2014), the luminally located aortic endothelial cells were identified, and then caveolae in the endothelial cells were defined as apical or basal invaginations open to the surface.

### Statistical Analysis

All statistical analyses were performed using GraphPad Prism Software. Data were drawn to analyze after being tested for and meeting normality using the Kolmogorov-Smirnov test. Data comparisons between groups repeatedly over time were analyzed by two-way repeated-measures ANOVA followed by Sidak’s *post-hoc* test for multiple comparisons or with two-tailed, unpaired Student *t*-test, as appropriate. Data are expressed as mean ± SEM. *P* < 0.05 was considered statistically significant.

## Results

### Role of Caveolae in Human Arteriole Dilation in Patients With Type 2 Diabetes

Patient demographics and clinical data are presented in Table 1. Patients with or without T2D had similar age, gender, underlying diseases, and medications but exhibited increased glucose levels while on antidiabetic medication (Table 1). Note that the majority of patients were hypertensive and obese in this consecutive cohort.

**TABLE 1:** Patient demographics, diseases, medications, and procedures.

Variable	Non-diabetic	T2D
N	5	5
Men/Women	4/1	4/1
Age (years)	65 ± 4	71 ± 4
Height (cm)	168 ± 4	176 ± 6
Weight (kg)	86 ± 4	95 ± 20
Systolic blood pressure	146 ± 7	137 ± 6
Diastolic blood pressure	66 ± 8	69 ± 5
Serum glucose (mg/dL)	107 ± 6	135 ± 18*
**Underlying disease**		
Type 2 diabetes	0	5
Hypertension	5	4
Hypercholesterolemia	5	4
Coronary artery disease	3	2
Peripheral vascular disease	1	0
Valve disease	2	2
**Medications**		
ACE inhibitor	1	4
Angiotensin receptor blocker	3	1
Aspirin	4	3
Statins	4	3
Oral antidiabetics	0	5
Beta blocker	4	4
Diuretics	2	1
Calcium channel blockers	3	1
Clopidogrel	1	1
**Surgical procedures**		
Coronary artery bypass graft	4	4
Valve replacement	1	2

Data are means ± SEM; N, number of patients studied. *Indicates significant difference compared to non-diabetic patients.

**FIGURE 1:**
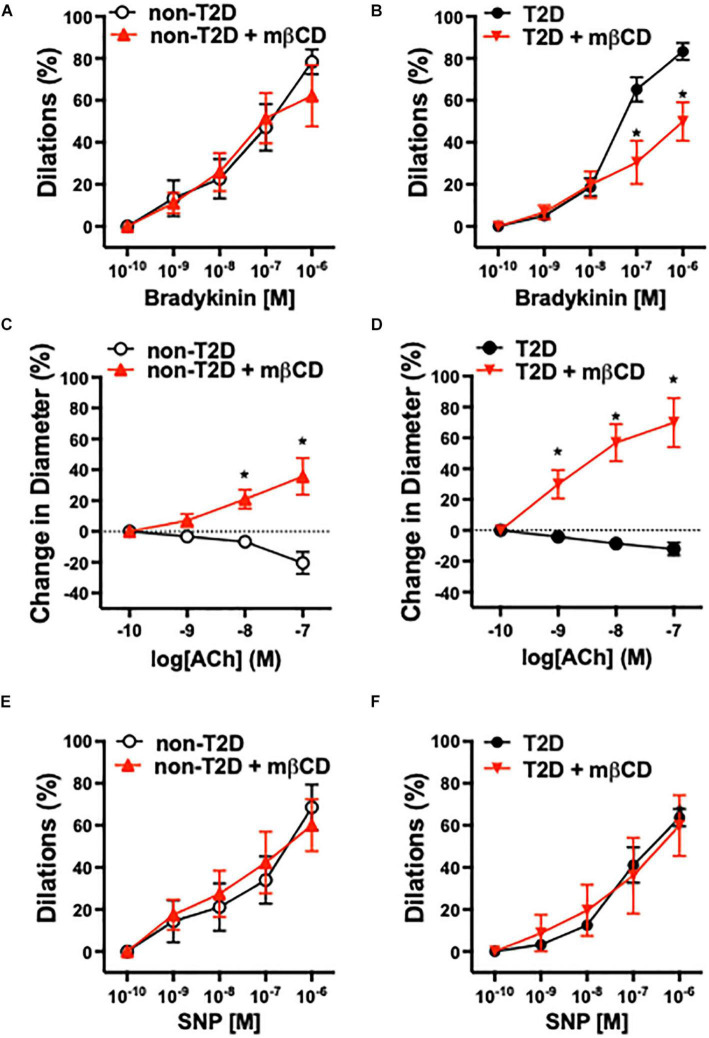
Role of caveolae in human arteriole dilation in patients with T2D. Summary data of percent diameter changes of atrial coronary arteries from non-diabetic (*n* = 5) **(A,C,E)** and T2D patients (*n* = 5) **(B,D,F)** in response to cumulative concentrations of bradykinin (10^–10^ to 10^–6^ M), acetylcholine (ACh, 10^–10^ to 10^–7^ M) and the NO donor, sodium nitroprusside (SNP, 10^–10^ to 10^–6^ M), before and after incubation with methyl-β-cyclodextrin (mβCD, 2 × 10^–3^ M) for 60 min. *p < 0.05 before vs. after mβCD.

Previous studies have shown that in patients with cardiovascular diseases, including those with diabetes, atrial coronary arterioles develop substantial vasodilation in response to bradykinin, whereas arterioles constrict in response to acetylcholine (ACh) (Miller et al., 1998; Szerafin et al., 2006). In this study, we found that the baseline (in calcium containing Krebs solution) and passive (in calcium-free Krebs solution) inner diameter of isolated and pressurized coronary arterioles were similar in T2D and non-T2D patients (baseline diameters: T2D: 89 ± 9 μm, non-T2D: 86 ± 8 μm; passive diameters: T2D: 112 ± 10 μm, non-T2D: 124 ± 10 μm). Bradykinin-induced dilations and ACh-induced constrictions of coronary arterioles were similar in magnitude in patients with or without T2D (Figures 1A–D). Interestingly, we found that pharmacologic disruption of vascular caveolae with mβCD significantly reduced bradykinin-induced dilation in coronary arterioles only in T2D patients, whereas mβCD had no effect on vasodilation in non-diabetic subjects (Figures 1A,B). Moreover, mβCD converted ACh-induced constrictions to dilations, with similar magnitude in arterioles of T2D and non-diabetic patients (Figures 1C,D). Dilations in response to the nitric oxide donor, sodium nitroprusside was similar in the two groups and it was not affected by mβCD (Figures 1E,F). Exposure to mβCD did not significantly affect the baseline diameters of coronary arterioles in either group (diameters after mβCD: T2D: 97 ± 10 μm, non-T2D: 85 ± 8 μm).

**FIGURE 2:**
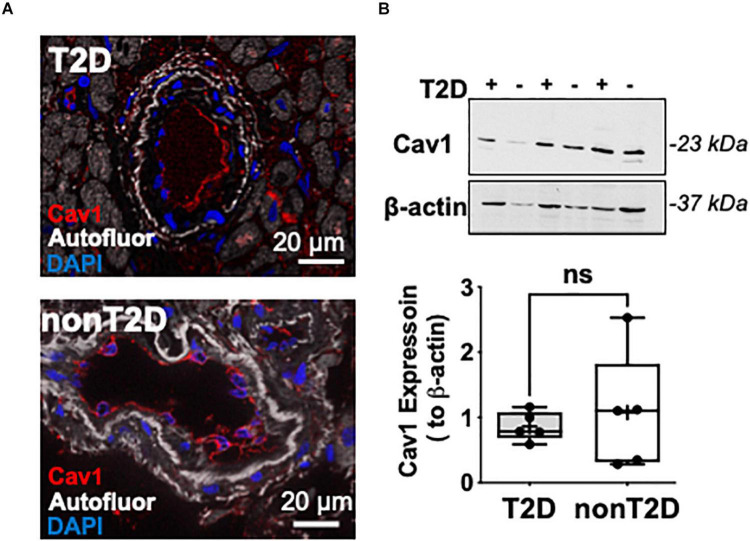
Representative microphotographs of immunofluorescent staining for Caveolin-1 (shown in red) in coronary arterioles from T2D and non-diabetic patients **(A)**. Nuclei were stained with DAPI (blue). Autofluorescence is shown in white. Scale bar = 20 μm. Representative images of Western immunoblot and summary data for protein expression of Cav-1 and β-actin in coronary arterioles obtained from T2D (*n* = 3) and non-diabetic (*n* = 3) patients **(B)**.

We also found that coronary arterioles abundantly express Cav-1, which is mainly localized to endothelial cells in both T2D and non-diabetic patients (Figure 2A). We did not find significant changes in the protein expression of Cav-1 between the two groups (Figure 2B).

### Role of Caveolae in Arteriole Dilation in Mouse Type 2 Diabetes Models, *ex vivo*

**FIGURE 3:**
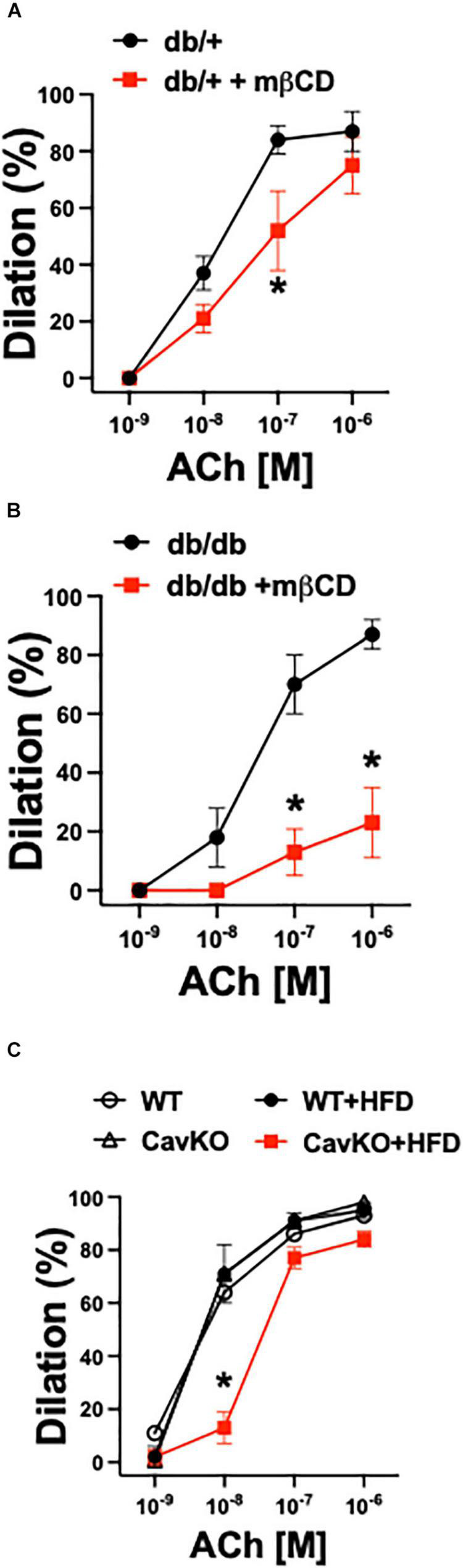
Role of caveolae in arteriole dilation in mouse T2D models, *ex vivo*. Summary data of percent diameter changes of skeletal muscle arterioles from non-diabetic, heterozygote control (db +) (*n* = 6–7) **(A)** and diabetic (db/db) (*n* = 6–7) **(B)** mice in response to cumulative concentrations of acetylcholine (ACh, 10^–9^ to 10^–6^ M) before and after incubation with methyl-β-cyclodextrin (mβCD, 2 × 10^–3^ M) for 60 min. *p < 0.05 before vs. after mβCD. **(C)** Summary data of percent diameter changes of skeletal muscle arterioles from normal chow diet or HFD fed Cav-1 knockout (CavKO, *n* = 4) and wild type (WT, *n* = 4) mice in response to cumulative concentrations of acetylcholine (ACh, 10^–9^ to 10^–6^ M). *p < 0.05 HFD CavKO vs. WT, Cav-1 KO and HFD WT mice.

Similar to our previous study (Bagi et al., 2005) obtained in isolated pressurized gracilis muscle arterioles, obese and hyperglycemic db/db mice had reduced baseline diameters when compared to controls (db/db: 67 ± 2 μm vs. db/ + : 81 ± 5 μm, *p* < 0.05), whereas the passive diameters were similar in the two groups (db/db: 128 ± 5 μm vs. db/ + : 130 ± 4 μm, non-significant). Also, the db/db mice had higher systolic and diastolic blood pressures and elevated glucose levels when compared to controls (Table 2). We found that mβCD reduced the endothelium-dependent, ACh-induced dilation in arterioles of db/db mice to a much greater extent than in non-diabetic controls (Figures 3A,B). Exposure to mβCD did not significantly affect the baseline diameters of arterioles in either group (diameters after mβCD: db/db: 65 ± 5 μm vs. db/ + : 76 ± 5 μm).

**TABLE 2:** Characteristics of db/db mice, wild type and caveolin-1 knockout mice with HFD.

Variable	Control	db/db
Body weight (g)	28.6 ± 1.6	60.2 ± 5.9*
Glucose (mM, fed state)	5.4 ± 0.4	17.6 ± 0.5*
Systolic blood pressure (mmHg)	118 ± 6	137 ± 7*
Diastolic blood pressure (mmHg)	72 ± 5	85 ± 4*

	**WT HFD**	**Cav-1 KO HFD**

Body weight (g)	57 ± 2	43 ± 3*
Glucose (mM, fed state)	11.3 ± 1.6	16.2 ± 0.9*
Systolic blood pressure (mmHg)	133 ± 3	151 ± 6*
Diastolic blood pressure (mmHg)	87 ± 3	107 ± 6*

*Indicates significant difference compared to control or wild type mice.

**FIGURE 4:**
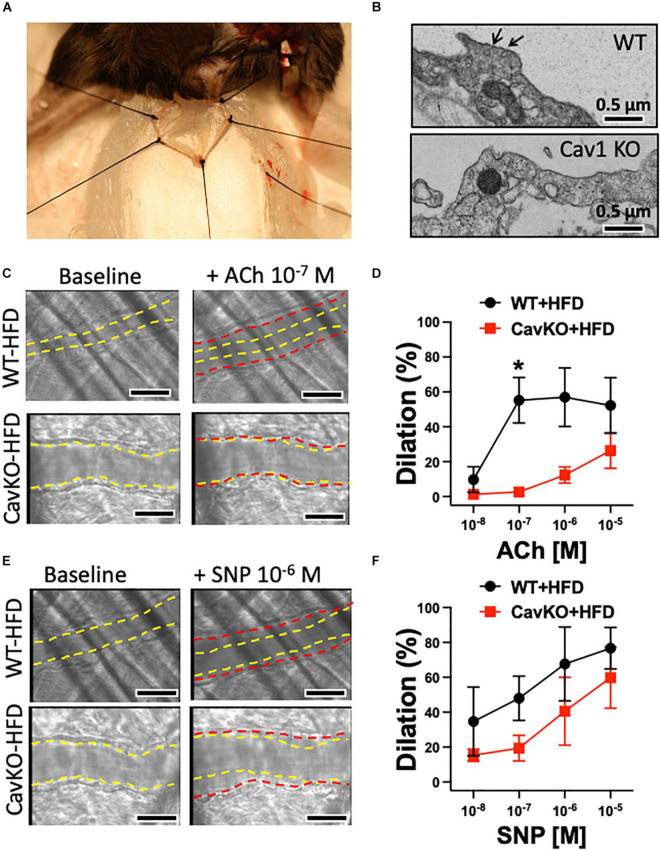
Dilations of the cremaster muscle arterioles in HFD fed Cav-1 knockout and WT mice, *in vivo*. Representative photo depicts the cremaster muscle preparation used for *in vivo* measurements of arteriole vasodilator function in the anesthetized mouse **(A)**. Representative electron microscopy photographs show caveolae (black arrow) in the aorta of WT mice and the absence of caveolae in the aorta of Cav-1 KO mice **(B)**. Scale bar = 0.5 μm. Representative original figures of baseline (yellow dashed line) and diameter changes (red dashed line) of cremaster muscle arterioles from HFD WT and Cav-1 KO mice in response to acetylcholine (ACh, 10^–7^ M) **(C)** or NO donor, sodium nitroprusside (SNP, 10^–6^ M) **(E)**. Scale bar = 20 μm. Summary data of percent diameter changes of cremaster muscle arterioles from HFD WT and CavKO mice in response to acetylcholine (ACh, 10^–8^ to 10^–5^ M) **(D)** or NO donor, sodium nitroprusside (SNP, 10^–8^ to 10^–6^ M) **(F)**. *n* = 4 in each group, *p < 0.05 HFD CavKO vs. HFD WT mice.

In addition to pharmacological disruption of caveolae, we also employed Cav-1 knockout and wild type mice that were fed either a normal chow diet or a high fat diet (HFD). Isolated gracilis muscle arterioles of Cav-1 knockout mice, that lack caveolae (Figure 4B), had similar baseline and passive diameters than wild type mice (baseline diameters: Cav-1 KO: 95 ± 6 μm, WT: 98 ± 5 μm; passive diameters: Cav-1 KO: 148 ± 5 μm, WT: 146 ± 6 μm), and developed similar vasodilation in response to ACh when on the normal chow diet (Figure 3C). While no significant changes were found in the baseline and passive arteriolar diameters (baseline diameters: Cav-1 KO + HFD: 94 ± 7 μm, WT + HFD: 99 ± 7 μm; passive diameters: Cav-1 KO + HFD: 148 ± 3 μm, WT + HFD: 137 ± 9 μm), the endothelium-dependent dilation to ACh was significantly reduced in HFD fed Cav-1 knockout mice when compared to HFD fed wild type mice (Figure 3C).

### Role of Caveolae in Arteriole Dilation and Serum Glucose in High-Fat Diet Fed Mice, *in vivo*

We also assessed endothelium-dependent vasodilator function, *in vivo*, in the mouse cremaster muscle (Figure 4A). The baseline initial (before ACh application) and maximally dilated (to 10^–4^ M SNP) inner diameter of cremaster muscle arterioles were similar in HFD fed WT and Cav-1 knockout mice (baseline diameters: WT + HFD: 26 ± 4 μm vs. Cav-1 KO + HFD: 28 ± 2 μm; maximal diameters: WT + HFD: 35 ± 3 μm vs. Cav-1 KO + HFD: 33 ± 2 μm). We found that ACh elicited substantial dilation in the cremaster muscle arteriole in HFD fed WT mice, however, dilation in response to ACh was significantly reduced in HFD fed Cav-1 knockout mice (Figures 4C,D). Dilation to the NO donor SNP shows a trend towards reduced dilation in Cav-1 knockout mice, but it did not reach significant difference (Figures 4E,F). Notably, we found that the serum glucose and blood pressure levels were elevated in HFD fed Cav-1 knockout mice when compared to HFD fed WT mice (Table 2).

## Discussion

Our study reveals that caveolae differentially regulate microvascular vasodilator function under the condition of hyperglycemia in T2D subjects. We also found that arterioles from diabetic subjects become more susceptible for caveolae disruption, which can exaggerate hyperglycemia in T2D. These conclusions were supported by the results from this study that mβCD, a caveolae disrupting agent, or genetic Cav-1 deficiency in mice impairs microvascular vasodilator function to a greater extent in patients and rodent models of T2D. We also found that HFD fed Cav-1 knockout mice, which lack caveolae, display a more pronounced vasodilator dysfunction and have elevated glucose levels, when compared to wild type HFD fed mice.

A solid line of evidence indicates that microvascular dysfunction interferes with insulin-mediated glucose uptake in animal models as well as in humans [reviewed in Muris et al. (2013)] and this, in part, can be attributed to the subsequent development of hyperglycemia in T2D subjects. Mechanistically, it has been proposed that vascular endothelium dysfunction influences the access of insulin to skeletal muscle cells, which requires adequate capillary perfusion (Lillioja et al., 1987; Serne et al., 1999; Sjoberg et al., 2017). It has also been reported that insulin can act as an endothelium-dependent arteriolar dilator (Laakso et al., 1990), which itself can augment capillary recruitment to further facilitate insulin-mediated glucose uptake, and this can be inhibited in obese subjects. Interestingly, a meta-analysis by Muris et al. revealed that impairment of microvascular function is independently associated with an increased incidence of T2D (Muris et al., 2012). The authors of this paper concluded that the association between microvascular dysfunction and hyperglycemia in T2D is likely to be bidirectional: hyperglycemia causes microvascular dysfunction and microvascular dysfunction precedes, and later exaggerates hyperglycemia. The nature of the underlying microvascular mechanisms through which arteriolar vasodilator dysfunction contributes to hyperglycemia and its progression remain incompletely understood in T2D.

Cell membrane caveolae uniquely regulate physiological functions, including microvascular mechanisms that control tissue perfusion and metabolism. In this process, previous studies have found that Cav-1, a key and essential protein for caveolae structure, plays an important role in the control of resistance artery vasodilator function *via* interacting with endothelial nitric oxide (NO) synthase (Venema et al., 1997) and calcium-activated potassium channels, BK(Ca) and SK(Ca), that mediate the endothelium-dependent hyperpolarizing factor (EDHF) response (Feher et al., 2010; Rath et al., 2012; Absi et al., 2019). More recently, we found a direct inhibitory interaction between Cav-1 and type 1 angiotensin receptor, which prevented a sustained angiotensin 2-induced constriction in skeletal muscle resistance arteries (Czikora et al., 2015). Based on these studies, we proposed that due to the unique interaction of Cav-1 with key molecular mediators of endothelium- and smooth muscle-dependent regulators of vasomotor function, caveolae provides protection against the development of elevated vascular resistance and increased systemic blood pressure. The current study extends this line of research and raises the possibility that caveolae also may play a role in glucose homeostasis and the manifestation of hyperglycemia, likely by affecting the microvascular vasodilator function in T2D subjects.

Results from the present study show that caveolae disruption has a greater influence on bradykinin-induced, likely EDHF-mediated, arteriole dilation in T2D patients when compared to non-diabetics. This data suggests a caveolae-dependent protection of vasomotor signaling in diabetes, which can serve as a compensatory mechanism to maintain tissue perfusion and metabolism, including glucose uptake. In this context, a previous study by Phillips et al. has shown that an impaired NO-mediated vasodilation is entirely compensated by an augmented, hydrogen peroxide-mediated EDHF response in diseased human blood vessels (Phillips et al., 2007). Interestingly, in this study we also found that caveolae disruption *via* exposure to mβCD, caused the human coronary arteriole constriction to ACh to be converted to a vasodilatory response. This can imply that the previously described inhibitory interaction between Cav-1 and endothelial NO synthase, limited the NO-mediated response (Venema et al., 1997). These aforementioned observations collectively suggest that (1) caveolae plays a unique role in facilitating EDHF-mediated arteriole dilation selectively in T2D, and (2) caveolae may also be involved in inhibiting NO signaling, which is similar in patients with or without T2D. How endothelial caveolae contributes to a selectively upregulated EDHF response is not well understood. In this context, caveolae disruption through exposing endothelial cells to oxidized LDL, cyclosporine or mβCD was shown to displace endothelial NO synthase from the plasma membrane (Blair et al., 1999; Lungu et al., 2004; Maniatis et al., 2006). We have shown previously that Cav-1 limits the contribution of BK(Ca) channels to the EDHF response, and that disruption of caveolae can lead to redistribution and greater contribution of the BK(Ca) channel to EDHF-mediated vasodilation (Feher et al., 2010). Moreover, we found earlier that in db/db mice there is an increased production of reactive oxygen species, including superoxide anion and hydrogen peroxide (Erdei et al., 2007). Hydrogen peroxide is a potent activator of calcium-activated potassium channels, including BK(Ca), SK(Ca), and IK(Ca), which can augment the EDHF response, and therefore have been even considered as therapeutic targets (Feletou, 2009). It is important to note, however, that this hydrogen peroxide-mediated compensatory vascular response may fail as T2D progresses, and excess hydrogen peroxide causes microvascular or cardiomyocyte impairments. In this context, microvascular dysfunction and hyperglycemia have been proposed to constitute a vicious cycle to yield impairments in organ function in T2D (Muris et al., 2012). We have also found previously that in diabetic patients the microvascular function becomes progressively compromised due to an excess production of reactive nitrogen species mainly driven by excessive hyperglycemia (Cassuto et al., 2014). We reported that Cav-1 is likely to undergo an increased 3-nitration by peroxynitrite, which could lead to destabilization of endothelial caveolae in T2D (Cassuto et al., 2014). There are a total of four tyrosine amino acids within Cav-1 protein that can be potentially nitrated. It is plausible that these modifications of Cav-1 alter protein binding partners and also destabilize caveolae and have an impact on its regulatory role. Therefore, it is possible that the observed adaptive vasodilator response by caveolae is temporary and highly susceptible to impairments if the causative factor, hyperglycemia, is not mitigated.

Another clinically relevant implication for this study is the unexpected detrimental effect of mβCD on arteriolar function observed selectively in T2D subjects. Cyclodextrins, α-, β-, and γ-cyclodextrins, have been exploited in the past as pharmaceutical probes and drug carriers, due to their physicochemical characteristics to form an inclusion complex with cholesterol and a variety of drug molecules (Uekama and Otagiri, 1987). Cyclodextrin itself has been considered as a potentially effective therapeutic agent for the treatment of atherosclerosis (Mahjoubin-Tehran et al., 2020). Several newly designed drug formulation complexes incorporating cyclodextrins as drug carriers have demonstrated a better efficiency in treating cardiovascular and neurodegenerative diseases (Coisne et al., 2016). Moreover, the efficacy of mβCD complexed with insulin is being evaluated in preclinical diabetes models (Sajeesh et al., 2010). Based on the results from our study, the development and use of mβCD in T2D subjects may interfere with caveolae dependent compensatory mechanisms and require further evaluation for microvascular effects and safety.

### Limitations

Our study has several methodological and mechanistic limitations. Due to human tissue availability and technical considerations working with mouse coronary microvessels, vasodilator function was assessed in T2D patients in the coronary microvessels, whereas rodent T2D models investigated skeletal muscle arteriole vasomotor responsiveness. It is known that the human coronary arteriole response to bradykinin is mainly mediated by EDHF, whereas the acetylcholine-induced response is linked to NO production. In the rodent skeletal muscle arterioles the response to acetylcholine is mainly mediated by EDHF. The contribution of NO, hydrogen peroxide, and EDHF to the altered vasoreactivity after caveolae disruption was not mechanistically evaluated in this study. As mentioned above, we reported earlier that the contribution of BK(Ca) channels to the EDHF response is more prominent after caveolae disruption by mβCD or in Cav-1 knockout mice (Feher et al., 2010). Nevertheless, there could be differences in specific vascular beds, coronary vs. skeletal muscle, regarding the mediators of EDHF and their caveolae dependency in T2D, which has yet to be elucidated. In this study we used mβCD to disrupt caveolae by removing membrane cholesterol. Another limitation arising from the reduced cell membrane cholesterol content, which independent from that of caveolae itself, is the possible effect on ion channel function, which should be considered. Of note, the percentage of cholesterol in caveolae/lipid rafts compared to total endothelial plasma membrane cholesterol levels is relatively high (Maxfield, 2002), therefore the cholesterol removal through use of mβCD mainly effects caveolae. We also observed that there was a trend toward reduced spontaneously developed tone in the cremaster muscle arterioles of the Cav-1 knockout mice. On the other hand, db/db mice had an increased spontaneously developed arteriolar tone, as we found earlier (Erdei et al., 2007). Changes in the spontaneously developed arteriolar tone can be attributed to alterations in the vascular smooth muscle cell function, both in the db/db and Cav-1 knockout mice, and this may contribute to the observed changes in percent dilations in these experiments. Moreover, insulin and glucose tolerance tests were not conducted in the rodent T2D models and Cav-1 knockout mice. It has been reported previously that Cav-1 knockout mice display insulin resistance and when fed a HFD, Cav-1 knockout mice develop postprandial hyperinsulinemia (Cohen et al., 2003). While vasodilator dysfunction in the skeletal muscle has been reported to interfere with glucose uptake both in animal models and in patients, skeletal muscle glucose uptake was not assessed in this study.

Taken together, our study demonstrates that subjects with T2D the role of caveolae in regulating endothelium-dependent arteriole dilation is altered and arterioles display a caveolae dependent, maintained vasodilator function which can mitigate or delay the development of hyperglycemia, likely *via* a mechanism which preserves glucose uptake in the skeletal muscle. Our study reveals for the first time that in subjects with T2D caveolae differentially regulate microvascular vasodilator function, which is likely to be mediated by a compensatory increased EDHF response. Under hyperglycemia or conditions that cause caveolae disruption, such as cyclodextrins or nitrosative stress, microvascular dysfunction can be exaggerated, and *via* an impaired tissue perfusion it can pose a significant risk for worsening hyperglycemia in patients with T2D.

## Data Availability Statement

The raw data supporting the conclusions of this article will be made available by the authors, without undue reservation.

## Ethics Statement

The studies involving human participants were reviewed and approved by Augusta University IRB. Written informed consent for participation was not required for this study in accordance with the national legislation and the institutional requirements. The animal study was reviewed and approved by Augusta University IACUC.

## Author Contributions

ZB conceptualized the project and supervised the research. ZB, YT, KF, VP, AF, and ZB performed the experiments. YT, KF, and ZB wrote, reviewed, and edited the manuscript. All authors contributed to the article and approved the submitted version.

## Conflict of Interest

The authors declare that the research was conducted in the absence of any commercial or financial relationships that could be construed as a potential conflict of interest.

## Publisher’s Note

All claims expressed in this article are solely those of the authors and do not necessarily represent those of their affiliated organizations, or those of the publisher, the editors and the reviewers. Any product that may be evaluated in this article, or claim that may be made by its manufacturer, is not guaranteed or endorsed by the publisher.
